# Integrating single-cell omics and materials science for uveal melanoma: from mechanistic insights to precision therapeutics

**DOI:** 10.3389/fonc.2025.1661889

**Published:** 2025-09-10

**Authors:** Shouyong Fu, Changfei Li

**Affiliations:** ^1^ Medical Device Department, Qingdao Hospital of University of Health and Rehabilitation Sciences, Qingdao, China; ^2^ Department of Ophthalmology, Qilu Hospital, Cheeloo College of Medicine, Shandong University, Qingdao, China

**Keywords:** uveal melanoma, medical device, nanotechnology, single-cell RNA sequencing, therapy

## Abstract

Uveal melanoma (UM), the most common primary intraocular malignancy in adults, presents significant clinical challenges due to its high metastatic potential, pronounced hepatic tropism, and poor prognosis upon systemic dissemination. Despite established local therapies, nearly half of patients develop distant metastases, highlighting an urgent need for more effective systemic strategies. Recent advances in single-cell omics technologies (e.g., scRNA-seq, scATAC-seq, spatial transcriptomics) have revolutionized our understanding of UM pathobiology. These approaches have meticulously delineated the complex tumor heterogeneity, immunosuppressive microenvironment, and key molecular drivers—including novel macrophage subsets (e.g., immunosuppressive MΦ-C4), senescent endothelial cells, and non-canonical immune checkpoint expression—providing unprecedented resolution for identifying actionable therapeutic targets. Concurrently, innovations in materials science and biomedical engineering offer transformative opportunities for precision therapy. Engineered nanocarriers, biodegradable implants, and advanced gene therapy vectors (e.g., tropism-enhanced AAVs, CRISPR-Cas9 systems) enabled targeted drug delivery, sustained release, and genetic modulation tailored to the eye’s unique anatomy and immune privilege. This review synthesizes these converging frontiers, outlining how the integration of multi-omics insights with smart biomaterials can overcome current therapeutic limitations. We catalog emerging material-based platforms applicable to UM and summarize validated molecular targets (e.g., GNAQ/GNA11, YAP/TAZ, BAP1, c-Met, CXCR4). Furthermore, we propose an interdisciplinary paradigm spanning combinatorial targeted therapies, immunomodulation, minimally invasive devices (e.g., robotic radiosurgery), and engineered delivery systems. By bridging mechanistic discovery with translational engineering, this synergy holds significant promise for advancing precision medicine and improving clinical outcomes in UM, ultimately facilitating the transition from bench to bedside.

## 1 Introduction

Uveal melanoma (UM), arising from melanocytes within the uveal tract, represents the most prevalent primary intraocular malignancy in adults. Approximately 90% of cases originate in the choroid, followed by 6% in the ciliary body and 4% in the iris (1). Unlike cutaneous melanoma, UM is characterized by distinct molecular features, including monosomy 3, chromosome 8q amplification (2, 3), recurrent mutations in GNAQ and GNA11, and an immunosuppressive (“cold”) tumor microenvironment (4, 5). These alterations underlie UM’s aggressive clinical course and strong hepatic tropism. Although UM accounts for only 3%–5% of all melanoma cases, its prognosis remains poor (6). In the United States, 1,700–2,500 individuals are affected annually (4.6–6 per million), with lower incidence among Black and Asian populations (0.3–0.4 per million) (7, 8). Nearly half of patients develop distant metastases after initial local therapy, with the liver involved in >90% of cases (9–11). Once systemic spread occurs, treatment options are limited and median survival falls below one year (12).

Local treatments—such as plaque brachytherapy, proton beam therapy, and enucleation—are well established but fail to prevent metastasis, highlighting the need for systemic strategies. Recent advances in materials science, particularly nanotechnology, have created transformative opportunities for ocular therapy. The eye’s anatomy and immune privilege favor localized delivery with high bioavailability and reduced systemic toxicity (13, 14). In UM models, poly(N-isopropylacrylamide) nanoparticles preferentially accumulated in uveal tissue (15), and polymeric or albumin-based nanocarriers delivering AZD8055 demonstrated selective cytotoxicity *in vitro* and *in vivo* (16). These innovations underscore the potential of engineered materials for precision ocular therapy. However, progress is hindered by the limited repertoire of actionable molecular targets, emphasizing the need for deeper understanding of UM pathology, particularly tumor heterogeneity and its immunosuppressive niche.

The advent of single-cell omics has revolutionized investigations into UM heterogeneity. Technologies such as scRNA-seq, scATAC-seq, and spatial transcriptomics provide unprecedented resolution for therapeutic target discovery (17–20). Recent studies have revealed unexpected clonal diversity, challenging the assumption that copy number variations are fixed early events (21). Li et al. identified a macrophage subset (MΦ-C4) associated with poor prognosis (22), while Tang et al. showed that infiltrating CD8^+^ T cells predominantly expressed LAG3 rather than PD1/CTLA4, explaining limited efficacy of conventional checkpoint inhibitors and suggesting LAG3 as an alternative target (23). Together, these findings illustrate how multi-omics approaches advance understanding of UM biology and inform novel therapeutic strategies.

This review synthesizes progress from single-cell omics and materials science, framing an interdisciplinary paradigm that integrates molecular discovery, engineered drug delivery, and clinical translation to advance precision medicine in UM.

## 2 Decoding UM heterogeneity: single-cell omics as a game-change

The emergence of single-cell technologies has profoundly transformed research on uveal melanoma (UM), providing unprecedented resolution to dissect tumor heterogeneity and therapeutic responses. Earlier bioinformatics approaches, relying on bulk sequencing and TCGA-derived signatures, enabled patient stratification and prognosis prediction but were limited by confounding noise and reductionist views of isolated genes (24). Single-cell analysis overcomes these constraints, capturing clonal dynamics and gene regulatory networks at the system level. This advance has fundamentally shifted mechanistic investigations and is now directly informing next-generation immunotherapies targeting checkpoint dysregulation (25–30). Unlike cutaneous melanoma, UM remains largely refractory to checkpoint blockade, highlighting the need to consider immune remodeling beyond T-cell–centric paradigms (31–33). Recent findings emphasize the roles of tumor-associated macrophages, senescent endothelial cells, and tumor-reactive lymphocytes, whose interactions create an immunosuppressive microenvironment resistant to conventional therapies.

### 2.1 Tumor-associated macrophages

Tumor-associated macrophages (TAMs) represent a critical cellular subset in UM, with their functional significance strongly linked to clinical outcomes. Accumulating evidence demonstrates that TAM infiltration correlates with UM-related mortality and established histopathological prognostic indicators, including the presence of epithelioid cells and elevated tumor microvascular density (MVD) (34). Notably, TAMs exhibit preferential accumulation in UM tumors harboring monosomy 3 karyotypes, which are characterized by an immunosuppressive microenvironment and inflammatory phenotypic features (35). Importantly, these TAMs predominantly display a pro-angiogenic M2-polarized macrophage phenotype, further potentiating tumor progression through vascular remodeling (36). Besides, Herwig et al. demonstrated that the imbalance between M1 and M2 macrophage polarization in UM is driven by PPARγ (37).

Beyond traditional chromosomal 3 analysis, contemporary prognostic frameworks integrate TAM-associated molecular signatures with advanced genomic tools. Gene expression profiling (GEP), for instance, enables stratification of UM into two distinct molecular classes: low-risk class 1 and high-risk class 2 tumors (38). Emerging data suggests that TAM-derived cytokines may synergize with class 2-specific genetic alterations to drive metastatic progression, positioning TAMs as both a biomarker and therapeutic target in UM management.

**Figure 1:**
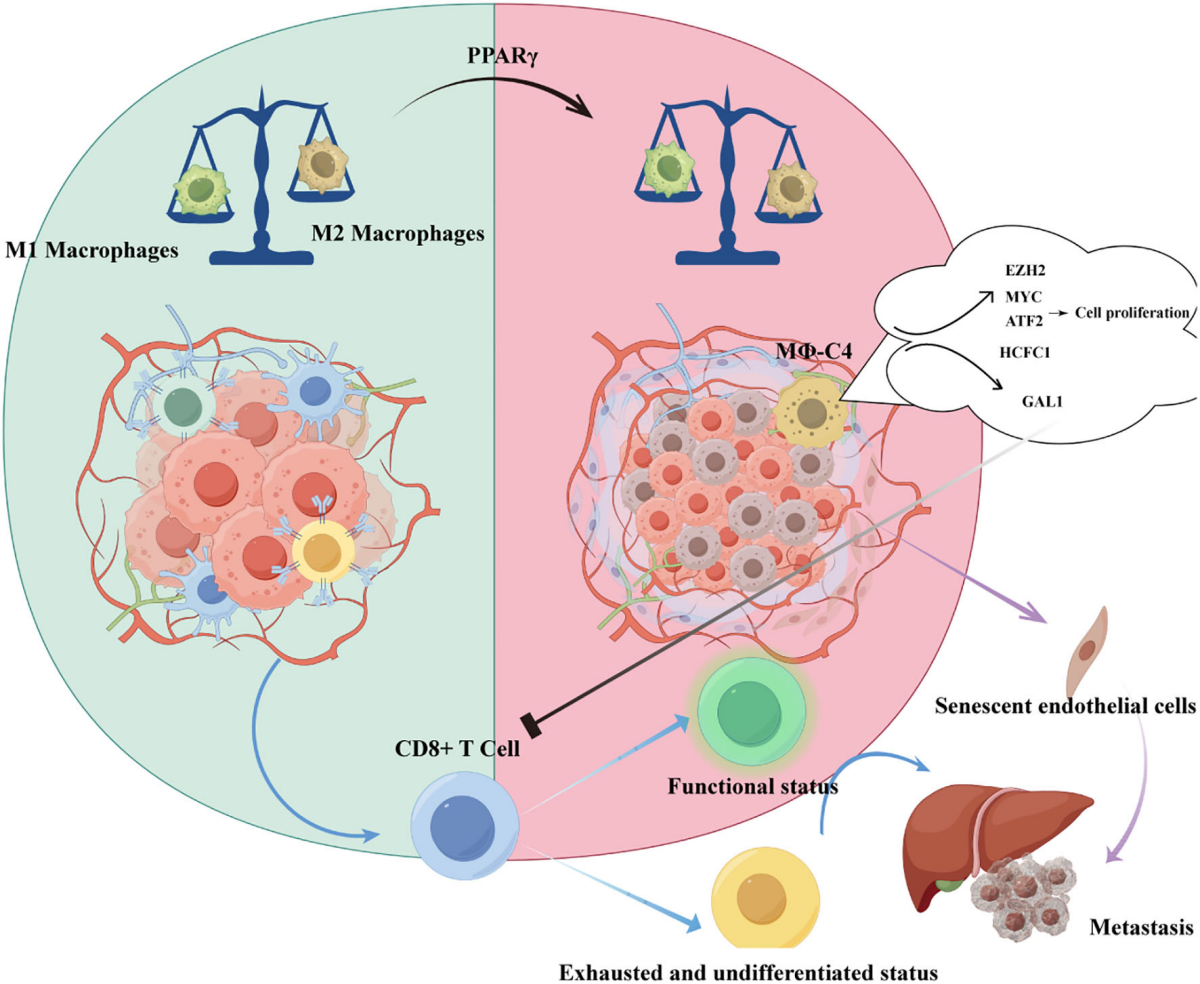
Tumor-associated macrophages and the immune microenvironment in uveal melanoma.

Recent single-cell transcriptomic analyses have refined macrophage classification beyond the conventional M1/M2 paradigm. Li et al. examined 63,264 single-cell transcriptomes from 11 UM patients and identified four transcriptionally distinct macrophage subsets (MΦ-C1 to MΦ-C4), confirming that MΦ-C4 serves as an independent prognostic factor (22). As illustrated in **Figure 1**, this subset promotes tumor cell proliferation and exerts immunosuppressive effects through representative regulatory pathways, underscoring its potential as a therapeutic target to overcome resistance to immune checkpoint blockade. Complementary work by Sun et al. defined a metastatic protective macrophage subpopulation (MPMφ) associated with antigen processing and inflammatory responses, which exhibited inhibitory effects on UM metastasis (39). Collectively, the refined classification of macrophage subpopulations significantly enhances understanding of UM’s immune landscape and informs the development of targeted diagnostic and therapeutic strategies.

### 2.2 Senescent endothelial cells

Endothelial cells (ECs) play pivotal roles in the tumor microenvironment, significantly influencing tumor development and progression. ECs form a monolayer lining the inner surface of blood vessels. Beyond regulating gas and metabolite exchange between vasculature and tissues, ECs modulate hemodynamics, coagulation, angiogenesis, and inflammation (40). Tumor growth critically depends on neovascularization to meet nutritional demands, while hematogenous metastasis relies on interactions with endothelial cells. Consequently, the status of vascular endothelial cells holds substantial implications, rendering endothelial cell injury a subject of considerable interest. Such injury may arise from factors including oxidative stress, inflammation, hyperglycemia, and senescence (41).

Zeng et al. demonstrated a remarkable disparity in endothelial cell (EC) abundance between primary and metastatic UM, with profoundly elevated ECs in metastatic UM exhibiting aberrant senescence. This senescent phenotype was attributed to the upregulation of KLF4, a pivotal senescence-associated transcription factor. Senescent ECs consequently secreted senescence-associated secretory phenotype (SASP) factors, which facilitate tumor cell recruitment and hepatic metastasis. Among SASP components derived from senescent ECs, the chemokine CXCL12 was identified as critical for mediating tumor cell migration induced by senescent endothelium (42).

### 2.3 Tumor-reactive lymphocytes

Tumor-reactive lymphocytes represent a pivotal component of the UM immune landscape. Recent work has refined the classification of T cell subpopulations within UM, offering novel strategies for selective expansion of tumor-infiltrating lymphocytes (TILs) to enhance both adoptive cellular therapy (ACT) and checkpoint blockade (43). These findings highlight that the efficacy of TILs may depend on reprogramming the tumor microenvironment from an immunologically “cold” state toward a pro-inflammatory niche that permits robust T cell infiltration. Such reprogramming may require adjunctive approaches, including lymphodepletion to reduce competition for interleukin-2 (IL-2) signaling and locoregional therapies that induce immunogenic cell death. For example, melphalan-based isolated hepatic perfusion (IHP) has been shown to trigger a surge of neoantigen-specific T cells, potentially priming previously non-inflamed tumor sites for immunotherapy responsiveness. In parallel, Chen et al. demonstrated that distinct CD8^+^ T cell states can exert profound influence on UM metastatic progression, underscoring the importance of targeting T cell dysfunction at multiple levels (44).

On the clinical front, monotherapy with immune checkpoint inhibitors (ICIs) has consistently demonstrated inferior efficacy in UM compared to metastatic cutaneous melanoma (32, 45). Combination regimens, such as PD-1/CTLA-4 inhibition (46–48) and PD-1/HDAC inhibitor strategies (49, 50), have shown encouraging improvements in survival relative to historical benchmarks (12). The most significant clinical advance to date is tebentafusp, a bispecific T cell engager targeting HLA-A2-positive individuals, which achieved the first survival benefit in a phase 3 trial, extending median overall survival from 16.0 to 21.7 months (51, 52). Locoregional therapies also remain clinically relevant: phase 3 trial data demonstrated that IHP tripled hepatic progression-free survival compared with best alternative care (53), although the subsequent SCANDIUM trial did not confirm a clear overall survival benefit (54). Nevertheless, ongoing clinical trials are actively investigating combinations of locoregional therapies with ICIs (NCT04463368) to exploit synergistic immunomodulatory effects. Importantly, both tebentafusp and IHP ultimately lead to disease progression, reinforcing the need for integrated strategies that combine biological insights into T cell dysfunction with innovative clinical interventions.

## 3 Ocular advanced materials: engineering precision delivery

With ongoing progress in precision medicine, ophthalmic treatments are steadily shifting toward strategies that are more targeted, effective, and minimally invasive. The incorporation of advanced material technologies has enabled novel approaches for ocular drug delivery, gene modification, and sustained-release therapies. These innovations are particularly promising for managing posterior segment disorders, such as retinal and choroidal diseases. This section offers a concise overview of several advanced materials used in ophthalmology, including nanocarrier systems, degradable implants, gene delivery vectors, and next-generation therapeutic platforms. We highlight recent developments in how these materials improve treatment specificity and longevity. Key functional features and representative research are summarized in **Table 1**.

**Table 1:** Material science-driven ocular precision drug delivery systems and their applications in UM.

Name	Material type / strategy	Mechanism of action	PMID
PEGylated nanostructured lipid carriers (NLCs)	Nanostructured lipid carriers (NLCs)	Sustained delivery of the prodrug (S)-(–)-MRJF22 to inhibit uveal melanoma growth, enhance biocompatibility, and enable topical delivery to the posterior eye segment	38851409
Cuprous oxide nanoparticles (Cu_2_O-NPs)	Inorganic nanoparticles	Internalized via lipid raft-mediated endocytosis; localize to mitochondria, lysosomes, and autophagolysosomes; induce ROS production, apoptosis, and autophagy; inhibit uveal melanoma proliferation, migration, and invasion	26467678
Hyaluronic acid nanoparticle carrying Verteporfin (HANP/VP)	Hyaluronic acid-based polymeric nanoparticle	Targets CD44-expressing UM cells; enables tumor-specific accumulation; enhances photodynamic therapy (PDT); inhibits YAP signaling; induces apoptosis and immune activation	38546166
Dexycu®	Biodegradable intraocular suspension (Verisome® technology	Single intraocular injection delivering dexamethasone for ~30 days post-cataract surgery; localized anti-inflammatory effect; reduces need for eye drops	36986595
Ozurdex®	Biodegradable PLGA-based intravitreal implant	Sustained dexamethasone release over months for treating retinal inflammation (e.g., DME, BRVO, CRVO, uveitis); inhibits cytokines; reinjected as needed	36986595
PCL-g-PDA [poly(ϵ-caprolactone)-graft-poly(dopamine)]	Biodegradable polyester copolymer	Mimics melanin drug-binding via PDA moieties; enables sustained intravitreal drug release through drug–catechol interactions; biocompatible and degradable	36170117
TM-loaded biodegradable subconjunctival microfilm	Biodegradable poly(lactide-co-caprolactone) (PLC) elastomer with PEG copolymers	Subconjunctival implant for sustained timolol maleate release over 3–5 months; reduces intraocular pressure; improves bioavailability and compliance	26100093
AAV-delivered Cre-mediated YAP/TAZ activation model	Genetic mouse model via AAV-Cre system	Demonstrates YAP/TAZ activation as sufficient for UM initiation; cooperates with Ras/MAPK in progression; dual inhibition shows synergistic antitumor effect	31801083
AAV-shRNA-mediated PCK1 knockdown	Gene therapy via AAV delivery	PCK1 promotes UVM cell growth via Gαi3-Akt signaling; silencing PCK1 inhibits proliferation, migration, and induces apoptosis in vitro and in vivo	38670942
PEI-DOCA modified cationic lipid nanoparticles (LNPs) delivering CRISPR/Cas9 RNPs	Functionalized lipid nanoparticle for gene therapy	Transdermal delivery of CRISPR/Cas9 RNPs targeting Braf gene; enhanced skin penetration and cellular uptake; in vivo genome editing leading to melanoma	39461690
ZAP-X® radiosurgery system	Novel stereotactic radiosurgery platform	Provides high-precision, single-session high-dose irradiation with real-time motion tracking; achieves tumor volume reduction in uveal melanoma	40291338
CP@Au@DC_AC50 hydrogel	Injectable stimuli-responsive hydrogel with gold nanorods	NIR-triggered photothermal therapy combined with controlled release of gene-targeted drug DC_AC50; antibacterial effect preventing ocular infection	34331418
Treat20 Plus precision oncology program	Precision oncology clinical workflow integrating multi-omics sequencing and multidisciplinary tumor board recommendations	Molecular profiling-guided matched therapies (MEK, MET inhibitors, checkpoint inhibitors); 60% of patients received targeted therapy with 56% clinical benefit rate	35569281

*Representative examples of nanocarriers, biodegradable implants, gene therapy vectors, medical devices, and clinical workflows developed for ophthalmic diseases and uveal melanoma. The table summarizes material types, mechanisms of action, and therapeutic relevance. NLCs, nanostructured lipid carriers; PLGA, poly (lactic-co-glycolic acid); PDA, poly (dopamine); PLC, poly (lactic acid-co-caprolactone); AAV, adeno-associated virus; LNPs, lipid nanoparticles; NIR, near-infrared.

**Table 1** was identified through systematic PubMed/Scopus searches (2018–2024), prioritizing entities meeting stringent criteria: (1) for targets, mechanistic validation in ≥2 *in vivo* uveal melanoma models (e.g., PDXs, GEMMs) AND clinical association (OS/PFS HR > 1.5 or phase I+ trial evidence); (2) for delivery systems, demonstrated ocular targeting in UM preclinical studies (tumor-to-liver ratio > 5:1) AND intraocular feasibility (e.g., vitreal half-life > 24h). Exclusion criteria encompassed purely *in vitro* data, non-peer-reviewed reports, and non-UM-specific platforms. Evidence was synthesized narratively but graded systematically (Level I: meta-analysis; Level II: RCTs; Level III: cohort studies), with acknowledged limitations in rapidly evolving fields (e.g., CRISPR-carrying exosomes post-2023). **Table 2** has the same search criteria as **Table 1**.

**Table 2:** Potential targets for treating UM.

Target	Mechanism of action	Targeted therapy/strategy	Research/clinical progress	PMID
GNAQ/GNA11 Mutations	Activates MAPK/ERK pathway, promoting tumor growth	MEK inhibitors (Trametinib, Selumetinib)	44% disease stabilization rate in clinical trials	35804836
YAP/TAZ Signaling Pathway	Promotes cell proliferation, migration, and survival	YAP inhibitors (Verteporfin), combined with Mcl-1 inhibition	Functional inhibition effective; combination therapy shows synergistic effect	31801083
BAP1 Gene Mutation	Associated with high malignancy and metastasis risk	Prognostic tool, no targeted drug available	BAP1 expression testing superior to traditional staging systems	38238977
c-Met	Facilitates cell migration and liver metastasis	c-Met inhibitors	Potential to inhibit UM metastasis; under investigation	36761417
CXCR4	Chemotactic migration to distant organs	CXCR4 antagonists	High expression linked to metastasis risk; effective inhibition observed	19553629
SF3B1 Mutation	Alters RNA splicing; associated with intermediate metastatic potential	Splicing modulation under study	Emerging target; ongoing research into its role in metastasis	35012916
MYC Pathway	Drives proliferation and metastasis; associated with monosomy 3	BET inhibitors (e.g., Mivebresib)	Common amplification; MYC-driven tumors show responsiveness to BET inhibition	11431428
Cell Cycle Regulators	Loss of CDKN2A; CDK4/6 activation leads to uncontrolled cell proliferation	CDK4/6 inhibitors (e.g., Palbociclib)	CDK pathway alterations common; CDK inhibitors under investigation	34924562
VEGF / Angiogenesis	Promotes angiogenesis and metastasis, especially to the liver	Anti-VEGF antibodies (Bevacizumab), multi-target inhibitors (Nintedanib)	Promising adjuvant role; limited effect on metastases; resistance possible; further trials ongoing	26761211
PRAME	associated with tumor progression, immune evasion, and poor prognosis	PRAME-directed immunotherapy (TCR-engineered T cells, vaccine-based approaches)	High expression associated with metastasis risk; immunotherapy under investigation	36794811

*Key molecular targets, associated mechanisms, therapeutic strategies, and clinical progress in uveal melanoma. UM, uveal melanoma; MAPK, mitogen-activated protein kinase; ERK, extracellular signal-regulated kinase; GNAQ, guanine nucleotide-binding protein subunit alpha q; GNA11, guanine nucleotide-binding protein subunit alpha 11; BAP1, BRCA1 associated protein-1; SF3B1, splicing factor 3b subunit 1; EIF1AX, eukaryotic translation initiation factor 1A X-linked; MEK, mitogen-activated protein kinase kinase; PKC, protein kinase C; YAP/TAZ, Yes-associated protein and transcriptional co-activator with PDZ-binding motif; PRAME, preferentially expressed antigen in melanoma; PMID, PubMed Identifier.

### 3.1 Nanoparticle-based drug delivery systems for posterior segment

Posterior segment ocular diseases, such as UM, age-related macular degeneration (AMD), and diabetic retinopathy, pose significant therapeutic challenges. These include the restrictive nature of ocular barriers, short drug half-life, and the risk of local adverse effects. In recent years, nanotechnology has rapidly advanced in ophthalmic drug delivery, emerging as a critical breakthrough in precision therapy for posterior segment disorders due to its small particle size, tunable surface functionality, and high biodegradability (15, 55).

Nanoparticles not only facilitate efficient trans-barrier delivery across the blood-retinal barrier but can also be engineered with specific ligands for active targeting, thereby enhancing drug accumulation at pathological sites. For example, Cimino et al. developed polyethylene glycol-modified lipid nanoparticles (PEG-LNPs) to deliver the prodrug (S)-(–)-MRJF22. This system enhanced the drug’s stability and sustained-release profile, achieved prolonged suppression of uveal melanoma cells, and demonstrated favorable posterior segment distribution and biocompatibility in animal models (56).

Moreover, the unique intracellular delivery mechanisms of nanomaterials have introduced novel avenues for antitumor therapy. Song et al. found that cuprous oxide nanoparticles (Cu_2_O-NPs) could selectively enter UM cells via lipid raft-mediated endocytosis, inducing mitochondrial and lysosomal damage and activating oxidative stress pathways, ultimately triggering apoptosis and autophagy while significantly suppressing cell proliferation and invasiveness (57).

In combination therapy strategies, the integration of targeted delivery with photodynamic therapy (PDT) shows promising synergistic effects. For instance, hyaluronic acid-coated, CD44-targeted nanoparticles (HANPs) delivering the photosensitizer verteporfin have been shown to enhance drug accumulation at the tumor site while simultaneously inhibiting the YAP signaling pathway and inducing immune activation. This dual mechanism offers precise targeting and immune modulation for UM treatment (58).

Researchers are further exploring multifunctional nanoplatforms that integrate drug delivery, imaging guidance, thermoresponsive release, and immunomodulation—forming intelligent theranostic systems. The development of such multimodal platforms provides a promising path toward personalized treatment of posterior segment diseases. However, nanoparticle-based therapeutic strategies still face multiple clinical translation barriers, including insufficient long-term safety evaluation, immune clearance, batch-to-batch production variability, and challenges in large-scale manufacturing (59).

Future research should focus on: (1) identifying disease-specific targets and refining vector modifications; (2) enhancing delivery efficiency and tissue penetration; (3) integrating imaging technologies for real-time monitoring; and (4) establishing robust multi-center clinical validation and commercialization pathways.

In summary, nanoparticle delivery systems offer unprecedented innovation in the treatment of posterior segment ocular diseases. They demonstrate substantial therapeutic potential, particularly in malignant conditions such as UM. With the continued convergence of materials science and biomedical engineering, personalized and programmable smart nanocarriers are poised to become a cornerstone of precision ophthalmic therapy.

### 3.2 Biodegradable implants for sustained therapeutic release

Effective management of posterior segment ocular diseases often relies on maintaining sustained and stable intraocular drug concentrations. However, conventional treatment methods such as intravitreal injections necessitate frequent administrations and are associated with complications including elevated intraocular pressure, intraocular infections, and retinal detachment (60). To overcome these limitations, biodegradable implants have emerged in recent years as promising long-acting drug delivery platforms. These systems can provide continuous drug release without repeated interventions, thereby improving patient compliance and therapeutic outcomes (61).

Such implants are commonly composed of biodegradable polymers including polylactic acid (PLA), poly (lactic-co-glycolic acid) (PLGA), and polycaprolactone (PCL). The degradation rate and drug release kinetics can be finely tuned according to clinical requirements (62, 63). Implants may be placed in various ocular compartments such as the vitreous cavity, sub-scleral space, or episcleral surface, typically via surgical or minimally invasive procedures. During degradation, they steadily release anti-inflammatory, anti-angiogenic, or anti-tumor agents (64).

In the treatment of UM, a primary malignant tumor of the posterior segment, no standardized biodegradable implant system has yet been widely adopted in clinical practice. Nevertheless, several studies have explored their potential in localized sustained drug delivery. For example, researchers have developed PLGA-based microspheres or nanofibrous membranes loaded with antineoplastic agents, which are implanted episclerally to deliver drugs directly to peritumoral tissues. This approach minimizes systemic toxicity and enables continuous tumor suppression (65). Other investigations have encapsulated photosensitizers or small-molecule targeted drugs within biodegradable carriers, achieving controlled release while enhancing drug concentration and specificity at the tumor site. These findings underscore the feasibility of such systems as novel local therapies for ocular tumors (15). Despite these advances, current research remains largely confined to animal models and early-phase clinical evaluations, and further efforts are required to facilitate clinical translation (66).

More broadly, the clinical application of biodegradable implants in posterior segment diseases has seen significant progress. For instance, Dexycu^®^ (a dexamethasone intraocular implant) and Ozurdex^®^ (a PLGA-based sustained-release dexamethasone system) have been approved by the U.S. FDA for the treatment of intraocular inflammation and macular edema, demonstrating favorable efficacy and safety profiles (61, 67). Furthermore, Bahuon et al. developed a novel polyester copolymer (PCL-g-PDA) containing poly(dopamine) (PDA), which mimics natural ocular melanin and enhances drug-binding capacity. This system successfully extended the release duration of dexamethasone and ciprofloxacin, showing promise as a biodegradable vitreous implant (68). Another study introduced a degradable microfilm based on poly (lactic acid-co-caprolactone) (PLC) elastomers for the sustained delivery of timolol maleate (TM) via sub-scleral injection. By integrating a multilayer structure with PEG modifications, this system achieved stable drug release for over three months and demonstrated excellent intraocular pressure-lowering effects and biocompatibility in non-human primate models (69).

In summary, biodegradable implants offer a transformative strategy for the treatment of posterior segment diseases by enabling long-term, stable, and controlled drug delivery. They are particularly well-suited for chronic conditions such as UM that require prolonged management. Looking ahead, advancements in multi-drug co-delivery, disease microenvironment-responsive mechanisms, and minimally invasive implantation techniques are expected to further enhance the precision and clinical applicability of these systems.

### 3.3 Gene therapy vectors with ocular tropism

Gene therapy has emerged as a precise therapeutic strategy targeting the molecular mechanisms of disease, achieving remarkable progress in ophthalmology in recent years. The eye, with its relatively enclosed anatomical structure, strong immune privilege, and high bioavailability, is considered one of the most ideal target organs for gene therapy. In particular, the development of gene vectors with high tissue specificity, efficient delivery, and excellent safety profiles for ocular tissues—especially the retina and choroid—has become a major research focus (70).

Among current gene delivery systems, adeno-associated virus (AAV) vectors are the most widely used in ophthalmology due to their low immunogenicity, stable gene expression, and ability to target various intraocular cell types. Different AAV serotypes (e.g., AAV2, AAV8, AAV9) exhibit distinct affinities for retinal pigment epithelial cells, retinal ganglion cells, and choroidal endothelial cells, allowing for tailored targeting based on specific cell types (71, 72). The tropism and delivery efficiency of AAVs are critical factors in ocular gene therapy. Certain serotypes, such as AAV2, AAV5, and AAV8, show strong tropism toward retinal pigment epithelial and photoreceptor cells, making them preferred vectors for treating inherited retinal diseases (IRDs), such as Leber congenital amaurosis (LCA) and retinitis pigmentosa (RP) (73). Notably, Luxturna (voretigene neparvovec), an AAV2-based gene therapy delivering the RPE65 gene, became the first FDA-approved gene therapy for LCA in 2017, marking a milestone in the clinical translation of AAV platforms in ophthalmology (74).

Currently, over 70 clinical trials involving AAV-mediated gene therapy are underway, targeting various pathogenic genes including CNGA3, RPGR, CHM, and ND4. These trials span a range of diseases such as RP, X-linked retinoschisis, choroideremia, and age-related macular degeneration (AMD) (71, 75–77). Clinically, AAVs offer several advantages, including low immunogenicity, non-integration into the host genome, and sustained expression in terminally differentiated cells like photoreceptors. However, limitations remain, such as restricted packaging capacity (~4.7 kb), suboptimal targeting specificity, and the risk of pre-existing neutralizing antibodies in some individuals (78–80). To address the challenge of large gene delivery, dual-vector systems have been developed, allowing the expression of oversized genes and expanding AAV applicability in complex ocular disorders (81, 82). Moreover, recent research focuses on identifying novel AAV variants with enhanced ocular tropism (e.g., AAV2tYF, AAV7m8) and optimizing administration routes—including intravitreal, subretinal, and suprachoroidal injections—to further improve efficacy and safety (83, 84).

In the context of UM, AAV has served as a valuable tool for disease modeling and therapeutic exploration. AAV-mediated gene manipulation enables highly specific genetic control in uveal melanocytes, facilitating precise modeling of tumor initiation and progression. For instance, AAV-Cre recombinase delivery was used to establish a UM mouse model, revealing that activation of the YAP/TAZ signaling pathway alone can drive tumorigenesis and synergize with Ras/MAPK signaling to accelerate progression. Subsequent experiments demonstrated that co-inhibition of YAP/TAZ and Ras/MAPK pathways significantly suppressed UM malignancy, suggesting a promising dual-target therapeutic strategy (85). In therapeutic research, AAVs have also been employed to deliver RNA interference molecules. One study delivered shRNA-expressing AAVs into UM xenografts to silence PCK1, a gene promoting UM proliferation via the Gαi3-Akt pathway, resulting in significant tumor growth inhibition *in vivo* (86). These studies highlight the multifaceted utility of AAV in both mechanistic investigations and therapeutic development for UM.

In addition to viral vectors like AAV, non-viral gene delivery strategies have also gained traction, offering new possibilities for gene editing and regulation. The CRISPR-Cas9 system, known for its high specificity and flexibility, has been used for correcting pathogenic mutations in various ocular diseases. In UM, researchers have explored lipid nanoparticle (LNP)-mediated delivery of CRISPR-Cas9 components to target and edit GNAQ oncogenic mutations. This approach achieved efficient and relatively safe genome editing *in vitro* and *in vivo*, demonstrating mutation specificity and tumor-suppressive efficacy (87). Similarly, exosomes—due to their natural origin, excellent biocompatibility, and ability to cross biological barriers—have emerged as promising carriers for nucleic acid and gene delivery (88). Studies have utilized exosomes derived from retinal or stem cells to deliver miRNAs, siRNAs, or CRISPR components, aiming to modulate inflammation, angiogenesis, and oncogene expression, with early results supporting their feasibility in posterior segment disease treatment (89, 90). Although these non-viral vectors remain in early stages of development, their potential advantages in precision, safety, and delivery versatility are progressively expanding the scope of ocular gene therapy.

In summary, AAVs, CRISPR-Cas9-loaded lipid nanoparticles, and exosome-based platforms offer unique strengths and are collectively reshaping the treatment landscape for posterior segment disorders, particularly malignant conditions such as UM. Moving forward, enhancing delivery efficiency, cellular specificity, and safety will be pivotal for the successful clinical translation of gene therapies.

### 3.4 Emerging medical devices and treatment evolution

UM, the most common primary intraocular malignancy, is traditionally managed through radiation therapy (e.g., iodine-125 plaque brachytherapy) and surgical excision. However, these conventional approaches remain limited in their ability to fully control the tumor, preserve visual function, and prevent long-term metastasis (91, 92). In recent years, the integration of advanced engineering materials, microsystem technologies, and precision medicine has introduced a range of innovative medical devices and therapeutic strategies for UM, offering more targeted and controllable treatment options.

Among these, the ZAP-X stereotactic radiosurgery platform, a novel self-contained and self-shielded robotic system originally developed for treating brain and head-neck tumors, has recently been applied to UM management. This system employs three-dimensional imaging to precisely define tumor volumes and irradiation targets, combined with vacuum fixation and real-time infrared monitoring, to deliver single-session non-invasive radiation therapy. It eliminates the need for implant surgery and extended hospitalization required by traditional plaque therapy. Clinical data show that the ZAP-X system can effectively reduce tumor volume, with tumor control rates comparable to conventional radiotherapy and globe retention rates ranging from 78% to 97.4%. Its anesthesia-free, outpatient-based treatment model significantly enhances patient comfort and safety, representing a major advancement in radiotherapeutic management of UM (93).

In drug delivery, researchers have developed injectable, stimuli-responsive antibacterial hydrogels incorporating gold nanorods for synergistic photothermal therapy (PTT) and gene-targeted therapy. Upon near-infrared (NIR) light activation, this multifunctional platform enables on-demand drug release and gentle photothermal ablation of tumor cells. Additionally, its inherent antibacterial properties help prevent postoperative intraocular infections such as endophthalmitis, offering a minimally invasive approach that integrates precision drug release, thermal ablation, and infection control (94).

In the field of precision oncology, high-throughput genomic sequencing and molecular profiling technologies have enabled molecularly matched therapies for metastatic UM (95). Multidisciplinary tumor boards utilize whole-genome, exome, and transcriptome data to recommend targeted therapies, including MEK inhibitors, MET inhibitors, and immune checkpoint inhibitors. Clinical studies have shown that approximately 60% of patients receiving molecularly matched therapies experienced partial response or disease stabilization, with significantly prolonged progression-free and overall survival. This biomarker-driven strategy enhances therapeutic specificity and efficacy, surpassing outcomes achieved with conventional chemotherapy or immunotherapy.

Furthermore, single-cell technologies have been widely applied to dissect tumor heterogeneity, immune microenvironment, and metastatic potential in UM (21). Through single cell sequencing and cellular isolation, researchers can identify drug-resistant tumor subpopulations, immune cell interactions, and circulating tumor cells. These insights facilitate the development of personalized therapeutic strategies and offer a rational basis for targeting resistance mechanisms and metastatic pathways, ultimately contributing to long-term disease control.

In conclusion, emerging medical devices and therapeutic technologies are continuously expanding the landscape of UM treatment, significantly enriching the therapeutic arsenal. These innovations not only improve tumor control and preserve visual function while minimizing complications but also offer new hope for managing metastatic disease. Collectively, they are propelling UM treatment toward greater precision, minimal invasiveness, and personalized care.

## 4 Synergy of omics and materials: from bench to bedside

### 4.1 Potential targets for treating UM

UM is the most common primary intraocular malignancy. Although its incidence is relatively low, its high metastatic potential and poor prognosis make early targeted intervention a persistent clinical challenge. In recent years, advances in omics technologies have gradually elucidated the molecular mechanisms underlying UM, providing a valuable theoretical foundation for the development of targeted therapeutic strategies. Several potential therapeutic targets have been identified, along with corresponding research progress, as summarized in **Table 2**.

#### 4.1.1 GNAQ and GNA11 mutations

Mutations in *GNAQ* and *GNA11* are found in over 80% of UM patients and represent the most common driver mutations in this malignancy. These mutations lead to aberrant activation of the MAPK/ERK signaling pathway, thereby promoting tumor initiation and progression (5). In response to this mechanism, MEK inhibitors—such as trametinib and selumetinib—have been extensively studied as targeted therapies that inhibit downstream components of this pathway. By blocking MEK1/2, key kinases in the MAPK/ERK cascade, these agents effectively disrupt signal transduction and suppress tumor cell proliferation. Clinical data indicate that among 27 patients who received molecularly matched therapies, 15 were treated with MEK inhibitors, achieving a disease stabilization (SD) rate of 44% (96).

#### 4.1.2 YAP/TAZ signaling pathway

The YAP/TAZ signaling pathway, as the downstream effector of the Hippo signaling cascade, plays a pivotal role in the initiation and progression of UM. YAP (Yes-associated protein) and TAZ (transcriptional coactivator with PDZ-binding motif) promote malignant progression by regulating key cellular processes such as proliferation, survival, and migration. Aberrant activation of this pathway is therefore considered a major oncogenic mechanism in UM (85).

Multiple studies have demonstrated that inhibiting YAP/TAZ function can effectively suppress UM cell growth and reduce metastatic potential. For instance, the drug verteporfin (VP) disrupts the interaction between YAP and its transcriptional partner TEAD, thereby inhibiting tumor cell proliferation (97). Moreover, combinatorial therapeutic strategies targeting YAP/TAZ alongside other oncogenic drivers have shown synergistic effects. A notable example is the dual inhibition of YAP/TAZ and the anti-apoptotic protein Mcl-1, which significantly enhances suppression of UM cell survival (98).

Currently, the development of YAP/TAZ-targeted inhibitors is ongoing, including small molecules that block TEAD function or modulate YAP/TAZ activity through alternative mechanisms. These emerging agents hold promises as effective therapies not only for UM but also for a broad range of solid tumors.

#### 4.1.3 BAP1 gene mutations

BAP1 (BRCA1-associated protein 1) is a critical tumor suppressor gene, and its mutation is strongly associated with the aggressiveness and metastatic risk of UM (99). Studies have shown that loss of BAP1 protein expression or functional inactivation correlates with significantly reduced survival in UM patients. BAP1 mutations are frequently accompanied by monosomy 3, a chromosomal alteration that is a well-established predictor of metastasis in UM (100, 101).

Importantly, BAP1 mutations often arise during the early stages of tumorigenesis and are associated with larger tumor size and a markedly increased risk of metastasis. UM tumors harboring BAP1 mutations exhibit greater invasiveness and metastatic potential, leading to significantly shortened metastasis-free survival—averaging approximately 2.4 years (102). In addition to genetic alterations, the methylation status of the *BAP1* gene has also been identified as a crucial prognostic biomarker. Higher levels of *BAP1* promoter methylation are linked to poorer clinical outcomes (103).

Immunohistochemical detection of BAP1 protein expression has become an effective clinical tool for prognostic assessment in UM. Notably, it has demonstrated superior predictive value for overall survival (OS) compared to conventional staging systems and chromosomal analyses (100). Thus, *BAP1* mutations represent not only a key molecular mechanism underlying UM malignancy and metastasis but also a valuable basis for personalized treatment planning and risk stratification.

#### 4.1.4 Metastasis-associated pathways: c-Met and CXCR4

UM is characterized by a high metastatic potential, with a strong predilection for hepatic dissemination. Consequently, targeting metastasis-associated signaling pathways has become a key therapeutic strategy in UM. Among these, *c-Met* and *CXCR4* have been identified as critical molecular drivers of metastatic progression.

*c-Met*, the receptor for hepatocyte growth factor (HGF), plays a central role in promoting tumor cell migration, invasion, and metastasis. Aberrant activation of the c-Met signaling pathway has been closely linked to hepatic tropism in UM. Inhibitors targeting c-Met are currently undergoing clinical investigation and have shown promise in suppressing UM metastasis, making c-Met one of the most important anti-metastatic therapeutic targets (104, 105).

*CXCR4*, a chemokine receptor, contributes to the chemotactic migration of tumor cells and facilitates their dissemination to distant organs such as the liver. High expression of CXCR4 has been associated with an increased risk of metastasis in UM. Therefore, CXCR4 inhibition holds potential for disrupting the metastatic process (106).

Additionally, mutations in the *SF3B1* gene have been linked to intermediate metastatic risk. Aberrant splicing patterns resulting from these mutations may influence tumor dissemination behavior and represent an emerging area for targeted therapeutic development (107).

In summary, inhibitors targeting *c-Met* and *CXCR4*, along with ongoing investigations into the role of *SF3B1* mutations, currently form the core of molecular strategies aimed at preventing UM metastasis. These targeted approaches hold the potential to significantly improve both survival outcomes and quality of life for patients with UM.

The prognostic and therapeutic implications of key targets like BAP1 and SF3B1 exhibit nuanced context-dependency, necessitating integrated multi-omics frameworks to resolve discordant signals. For BAP1, spatial transcriptomics reconciles the paradox of genomic loss (strongly metastatic, HR > 5) coexisting with transcriptomic immune infiltration: CD8^+^T cells preferentially localize to BAP1-intact regions (*in situ* immune “hotspots”), while BAP1-null zones exhibit TGFβ-dominated fibrotic immunosuppression. This spatial segregation mandates a *hierarchical evidence rule*: genomic alterations > spatial microenvironment > bulk transcriptomics for metastasis risk stratification, though immune-rich BAP1-intact niches may retain susceptibility to checkpoint inhibitors. For SF3B1, single-cell multi-omics (scRNA-seq + scATAC-seq) deciphers heterogeneous outcomes: only mutations coupled with open chromatin at chr8q24 (*MYC* locus) or chr7q31 (*MET* locus) drive lethal metastasis via BRD9 mis-splicing and MET enhancer activation. Thus, a unified decision heuristic emerges: *Discordant oncogenic/immune signals are resolved by cross-validating (1) genomic alterations with epigenetic chromatin accessibility, and (2) bulk signatures with spatial cellular mapping to identify dominant biological drivers.

#### 4.1.5 Cell cycle regulators and the MYC pathway

Amplification of the *MYC* gene and dysregulation of cell cycle-related pathways are frequently observed in UM, and both contribute synergistically to tumor initiation and progression. More than 80% of UM patients exhibit *MYC* amplification, with expression levels positively correlated with tumor size. *MYC* promotes tumor aggressiveness by enhancing cell proliferation and metastatic potential, and is strongly associated with monosomy 3, suggesting it may function as an independent driver of tumor progression (108, 109).

Cell cycle abnormalities are also common in UM, including deletions of *CDKN2A* and amplifications of *CCND1/2/3* and *CDK6*. These genetic alterations disrupt normal cell cycle control, enabling unchecked cell division. The *CDKN2A* gene encodes the tumor suppressor proteins p16^INK4a and p14^ARF, which are critical regulators of the G1/S transition. Loss of *CDKN2A* function leads to sustained activation of CDK4/6, thereby facilitating uncontrolled tumor growth (110, 111).

Based on these molecular insights, CDK4/6 inhibitors (e.g., palbociclib) and BET inhibitors (e.g., mivebresib) have emerged as promising therapeutic candidates in UM (110, 112). CDK4/6 inhibitors can arrest aberrant cell cycle progression, while BET inhibitors suppress MYC transcriptional activity, thereby targeting MYC-driven UM. Combinatorial approaches that simultaneously target both the MYC and cell cycle pathways hold significant potential for developing personalized treatment regimens for UM patients.

#### 4.1.6 Anti-angiogenic therapy

The growth of UM is also dependent on tumor angiogenesis, with vascular endothelial growth factor (VEGF) and its receptors being highly expressed in UM. These factors play a pivotal role in promoting tumor progression and metastasis (113). VEGF is a key regulator of vascular permeability, endothelial cell proliferation, and migration. Its levels are significantly elevated in UM patients, particularly those with metastatic disease. Excessive VEGF expression not only facilitates neovascularization but also contributes to hematogenous dissemination of tumor cells, which is one of the leading causes of mortality in UM patients (114).

Bevacizumab, a humanized monoclonal antibody targeting VEGF, inhibits angiogenesis by blocking the binding of VEGF to its receptors, thereby suppressing endothelial cell proliferation and vessel formation. It has become a widely used anti-angiogenic agent in the treatment of various malignancies, including melanoma (115). Although anti-angiogenic therapy is not yet a standard treatment for UM, and its efficacy in both primary and metastatic UM requires further clinical validation, existing studies suggest it holds potential as an adjuvant therapeutic option (116). In addition, novel multi-targeted inhibitors such as nintedanib have demonstrated inhibitory effects on primary UM cells, though their efficacy against metastases remains limited (117).

A major current challenge is the potential for resistance to VEGF-targeted monotherapy. Future strategies may involve combination regimens that integrate anti-angiogenic agents with other modalities—such as immunotherapy or PARP inhibitors—to enhance overall therapeutic efficacy.

Overall, anti-angiogenic therapy represents a promising avenue for UM treatment, though broader clinical application will require further substantiation through well-designed trials.

#### 4.1.7 PRAME antigen

Preferentially expressed antigen in melanoma (PRAME) has recently gained attention as a highly relevant immunotherapeutic target in UM (118). PRAME is a cancer-testis antigen that is minimally expressed in normal adult tissues but frequently upregulated in UM, particularly in high-risk tumors associated with monosomy 3 and BAP1 loss [36788079]. Elevated PRAME expression correlates with poor prognosis and metastatic potential, positioning it as both a biomarker and a therapeutic candidate.

In UM, PRAME is expressed in a substantial subset of tumors, typically ranging from about one-quarter to nearly half of reported cases, and its presence consistently correlates with unfavorable clinical features. High PRAME expression has been associated with larger tumor size, advanced TNM stage, frequent chromosome 8q gain, and an inflammatory phenotype, all of which contribute to its prognostic value. Importantly, PRAME-specific T cell receptor (TCR)-transduced T cells have demonstrated the ability to selectively kill UM cells in preclinical studies, underscoring its therapeutic relevance (119). Collectively, these findings highlight PRAME not only as a biomarker of high-risk disease but also as a promising candidate for adjuvant immunotherapy in UM.

In conclusion, targeted therapy for uveal melanoma is rapidly evolving toward multi-targeted and personalized approaches. Omics-based insights have highlighted biomarkers such as *GNAQ/GNA11*, *BAP1*, *MET*, *MYC*, and high tumor mutational burden (TMB) as critical foundations for precision medicine. Future therapeutic strategies are likely to integrate multiple targeted agents with immunotherapies, offering expanded treatment horizons and improved outcomes for patients with UM.

### 4.2 Potential ways of integrating medicine and engineering

With the ongoing convergence of medicine and engineering, particularly by advances in precision medicine and biomedical technologies—interdisciplinary integration has emerged as a vital pathway for improving therapeutic outcomes and patient quality of life. In the treatment of UM, the integration of medical and engineering innovations is playing an increasingly pivotal role, fostering novel therapeutic strategies and advancing clinical interventions.

#### 4.2.1 Smart drug delivery systems

Drug delivery systems represent a key area of medicine–engineering integration, particularly in UM treatment, where precise drug targeting is essential for maximizing efficacy while minimizing side effects. Through micro- and nanotechnology, researchers have developed targeted drug delivery platforms that combine nanomaterials (e.g., nanoparticles, nanocapsules) with engineered liposomes to deliver anticancer agents or gene editing tools directly to tumor sites. These systems not only facilitate effective penetration across ocular barriers but also enable sustained and controlled drug release, thereby significantly enhancing the specificity and effectiveness of UM therapies (15, 16).

#### 4.2.2 Photodynamic therapy and optical engineering

Photodynamic therapy (PDT) is an effective approach for treating UM, based on the principle that photosensitizers produce reactive oxygen species (ROS) upon activation by near-infrared (NIR) light, leading to selective tumor cell destruction. In recent years, the integration of optical engineering and nanotechnology has led to the development of advanced photosensitizer carriers and laser delivery systems capable of precisely targeting tumor regions while minimizing damage to surrounding healthy tissues. For example, the combination of miniaturized laser devices with high-efficiency photosensitizers enables localized irradiation of the tumor without compromising retinal structure, thereby enhancing therapeutic outcomes (120).

Moreover, PDT has been shown to stimulate the immune response by inducing the secretion of pro-inflammatory and antitumor cytokines such as IL-6, IL-1, and TNF-α. This immune activation enhances systemic tumor control and reduces the risk of progression and metastasis (121). PDT has demonstrated safety and efficacy in treating small, pigmented UM lesions and is emerging as a minimally invasive, highly targeted therapeutic modality. Although current treatments remain limited in effectiveness—with approximately 50% of patients developing metastases, the advancement of multiphoton excitation techniques in PDT offers new therapeutic possibilities (122). Further studies in animal models and clinical trials are needed to develop specialized devices and translate PDT innovations into routine clinical practice.

#### 4.2.3 3D printing and personalized medicine

3D printing technology has become increasingly established in medicine and shows considerable promise in the treatment of UM. By creating patient-specific printed tumor models, researchers can simulate drug delivery and surgical resection strategies in a virtual environment, enabling the design of highly personalized treatment plans. These individualized 3D-printed tumor replicas allow for more accurate evaluation of therapeutic efficacy and assist clinicians with preoperative planning and risk assessment (123).

Additionally, the development of customized ocular implants using 3D printing offers UM patients more personalized therapeutic options. For instance, patient-specific radiotherapy plaques or structural supports can be fabricated to restore vision and ocular function, enhancing post-treatment quality of life (124).

#### 4.2.4 Artificial intelligence and imaging analysis

The integration of artificial intelligence (AI) and imaging technologies holds tremendous potential in the diagnosis and treatment of UM. Leveraging deep learning and convolutional neural networks (CNNs), AI can automatically analyze multimodal imaging data—including fundus photography, optical coherence tomography (OCT), and ultrasound—to accurately extract tumor features, thereby significantly improving diagnostic accuracy and efficiency (125). AI systems can assist in early screening, tumor localization, and morphological assessment, as well as play critical roles in radiotherapy planning and postoperative surveillance.

Moreover, by integrating pathological and genomic data, AI is driving the advancement of personalized medicine in UM. However, several challenges remain, including data scarcity, clinical integration, and model interpretability. With ongoing technological progress, the application of AI in imaging analysis is expected to become a routine tool in UM management, enhancing clinical decision-making and improving patient outcomes (126).

#### 4.2.5 Minimally invasive and robot-assisted surgery

As robotic technologies continue to advance, minimally invasive surgical approaches are becoming increasingly prevalent in the treatment of UM. Robot-assisted stereotactic radiosurgery (SRS) systems such as CyberKnife and ZAP-X offer high-precision, single-session, non-invasive treatments that eliminate the need for surgical implantation of radioactive plaques and prolonged hospitalization associated with traditional radiotherapy (93, 127).

These robotic systems utilize multimodal imaging (MRI, CT, ultrasound) to perform three-dimensional tumor localization and dose planning, ensuring accurate tumor coverage while preserving surrounding healthy tissue. Clinical studies have reported globe retention rates of 73% to 87% and local tumor control rates of approximately 70% to 90% at a median follow-up of 3 to 5 years, with most patients maintaining functional vision (128). In addition, robotic SRS treatments are typically performed on an outpatient basis, requiring only local anesthesia and avoiding craniotomy or enucleation, thereby greatly reducing patient discomfort and recovery time. The safety, efficacy, and patient tolerability of these systems have been well demonstrated, representing a significant shift toward minimally invasive, precise, and personalized UM therapy.

In summary, minimally invasive robot-assisted radiosurgery is becoming a mainstream option for UM treatment. Combining effective tumor control with ocular preservation, this approach offers patients a safer, more comfortable therapeutic experience and reflects the future direction of advanced ophthalmic oncology care.

#### 4.2.6 Biomaterials and tissue engineering

The integration of biomaterials and tissue engineering offers a novel direction for the treatment of UM. By utilizing 3D-printed or biodegradable biomaterials, it is now possible to fabricate personalized implants, such as structural supports for post-tumor resection or bioengineered scaffolds for regional tissue repair (129). Moreover, the combination of biomaterials with stem cell therapies facilitates ocular tissue regeneration and repair, providing UM patients with improved postoperative recovery options.

Practical applications include patient-specific 3D-printed implants, biodegradable scaffolds, stem cell carriers, and systems designed to modulate tissue regeneration via growth factors or microenvironmental cues (130). As materials science and regenerative medicine continue to advance, biomaterials and tissue engineering are expected to become integral components of UM treatment, driving progress toward precision and personalized care.

In summary, innovative medicine–engineering integration strategies have introduced new paradigms for the treatment of uveal melanoma. These technologies—including smart drug delivery systems, photodynamic therapy, 3D printing, artificial intelligence, robotic-assisted surgery, and tissue engineering—not only offer more effective and individualized treatment options but also align with broader movement toward precision, personalized, and minimally invasive oncology. As technological innovation and interdisciplinary collaboration deepen, medicine–engineering convergence is poised to deliver further breakthroughs in UM treatment, significantly improving patient survival and quality of life.

#### 4.2.7 Target-delivery synthesis matrix

Furthermore, we have added a dedicated section integrating multi-omics insights into dosing regimens. Building on previous work, **Table 3** provides recommended combination strategies for different targets.

**Table 3:** Target-delivery synthesis matrix.

Target category	Molecular target	Optimal delivery platform	Biological rationale	Anticipated translational hurdles
Oncogenic Signaling	GNAQ/GNA11	Ligand-targeted nanocarriers (e.g., CD44-HA NPs)	Membrane-bound GPCRs require rapid cytoplasmic delivery of small-molecule inhibitors (e.g., MEKi). Surface-modified NPs enhance trans-scleral permeability.	Limited tumor penetration in avascular regions; burst release toxicity.
Transcriptional Regulators	YAP/TAZ	Tropism-enhanced AAVs (e.g., AAV2tYF)	Nuclear localization necessitates sustained CRISPR-mediated gene editing. AAVs achieve long-term expression in uveal melanocytes.	Pre-existing neutralizing antibodies (∼30% patients); packaging capacity limits (<4.7 kb).
Spliceosome Mutations	SF3B1	pH-responsive lipid nanoparticles (LNPs)	Mutant SF3B1 drives cytoplasmic mis-splicing. LNPs deliver splice-switching oligonucleotides (SSOs) with efficient endosomal escape.	RNA payload degradation in vitreous; batch-to-batch variability.
Metastasis Drivers	c-MET/CXCR4	Implantable sustained-release systems (e.g., PLGA microfilms)	Chemokine receptors require continuous pathway blockade. Biodegradable implants maintain stable drug concentrations (>3 months).	Fibrotic encapsulation reducing drug diffusion; surgical implantation complexity.
Tumor Suppressors	BAP1	Dual AAV vectors	Functional restoration requires genomic integration of large cDNA sequences (∼4.8 kb). Dual-vector systems enable oversized gene delivery.	Immune clearance; risk of genomic insertional mutagenesis.
Immunomodulators	LAG3/PRAME*	Exosome-based platforms	Immune checkpoint proteins demand spatiotemporal modulation. Autologous exosomes evade clearance and deliver miRNAs/siRNAs to TAMs.	Scalable GMP production challenges; low payload loading efficiency.

## 5 Future expectation

The eye represents a highly specialized organ system where cutting-edge biomaterials demonstrate significant potential for treating related diseases. UM, one of the most critical ocular malignancies, still necessitates more advanced and effective first-line therapies. Recent advances in single-cell omics have meticulously delineated the tumor immune microenvironment and pathogenic mechanisms, offering unprecedented insights. The integration of these insights with emerging materials science presents a promising avenue to overcome the challenges of UM treatment. This review aims to contribute to the advancement of therapeutic strategies for this disease. We first catalog material-based approaches applicable to UM therapy and summarize currently targetable key molecular pathways. Looking ahead, researchers in the field are encouraged to strategically combine these modalities to facilitate successful translation from laboratory findings to clinical applications.
